# Gender Differences in Sexually Active Life Expectancy in France

**DOI:** 10.1111/psrh.70076

**Published:** 2026-09-03

**Authors:** Caroline Moreau, Lena Pamboutzoglou, Antoine Sireyjol, Nathalie Bajos

**Affiliations:** ^1^ Inserm—Soins Primaires et Prévention, CESP Centre for Research in Epidemiology and Population Health Villejuif France; ^2^ Department of Population, Family and Reproductive Health Johns Hopkins University Bloomberg School of Public Health Baltimore Maryland USA; ^3^ IRIS, Inserm/EHESS/CNRS Aubervilliers France; ^4^ Centre Max Weber (CMW) ENS de Lyon Lyon France

## Abstract

**Introduction:**

A life course perspective on sexual activity is gaining attention due to increased longevity and the relationship between health and sexual behavior. Building on Lindau's and Gavrilova's concept of sexually active life expectancy, we examine how including non‐partnered sexual behaviors and satisfaction influences sexual life expectancy by gender in France.

**Methods:**

We estimate sexually active life expectancy using Sullivan's method, which integrates life table data from the 2023 vital registration with sexual activity prevalence from the CSF‐2023 French national survey. We also calculate a quality‐adjusted sexually active life expectancy that accounts for sexual satisfaction. Finally, we identify sociodemographic and health factors related to having a satisfying sexual activite life among individuals aged 50–89.

**Results:**

A 20–24‐year‐old man has a sexually active life expectancy of 47.1 years, compared to 44.5 years for a woman. This figure expands to 52.9 years for men and 48.9 years for women when including masturbation. The gender gap narrows when considering sexual satisfaction, with men losing 11 years and women 8 years of sexual life expectancy due to poor sexual satisfaction. Among individuals aged 50–89, the gender gap in being sexually active and satisfied narrows from OR = 0.64 [0.57–0.72] after adjusting for age to OR = 0.76 [0.67–0.87] after further adjustments for partnership status, health, and sociodemographics.

**Conclusion:**

This study enhances population‐level indicators of sexual health by broadening the understanding of sexuality beyond partnered behaviors. It reveals significant gender differences in sexual experiences throughout the life course, which lessen when considering sexual quality.

## Abstract (fr)

**Introduction:**

Une perspective de parcours de vie sur l'activité sexuelle suscite un intérêt croissant en raison de l'allongement de la durée de vie et de la relation entre santé et comportements sexuels. En nous appuyant sur le concept d'espérance de vie sexuellement active de Lindau et Gravilova, nous explorons comment la prise en compte de la masturbation et l'expérience subjective, telles que la satisfaction sexuelle, influencent les projections d'activité sexuelle au cours de la vie selon le genre.

**Méthodes:**

Nous estimons l'espérance de vie sexuellement active en France en utilisant la méthode de Sullivan, qui combine les tables de mortalité de l'INSEE (2023) avec la prévalence de l'activité sexuelle par âge provenant de l'enquête nationale CSF‐2023. Nous calculons également une espérance de vie sexuellement active ajustée sur la satisfaction sexuelle. Par ailleurs, nous analysons les facteurs sociodémographiques et de santé liés à l'activité sexuelle jugée satisfaisante chez les individus âgés de 50 à 89 ans, en stratifiant les résultats par sexe.

**Résultats:**

Un homme âgé de 20 à 24 ans a une espérance de vie sexuellement active de 47.1 ans, contre 44.5 ans pour une femme du même âge. Ce chiffre passe à 52.9 ans pour les hommes et 48.9 ans pour les femmes si l'on inclut la masturbation. L'écart entre les sexes se réduit lorsqu'on considère la satisfaction sexuelle, les hommes perdant en moyenne 11 ans d'espérance de vie sexuelle, contre 8 ans pour les femmes. L'analyse de régression pour les individus de 50 à 89 ans montre un écart entre les sexes qui diminue dans l'activité sexuelle jugée satisfaisante passant d'un OR = 0.64 [0.57–0.72] après ajustement pour l'âge à OR = 0.76 [0.67–0,87] après ajustements supplémentaires pour le statut de partenariat, la santé et les caractéristiques sociodémographiques.

**Conclusion:**

Cette étude enrichit les indicateurs de santé sexuelle à l'échelle de la population en élargissant la compréhension de la sexualité au‐delà des comportements impliquant des partenaires et met en lumière des différences significatives entre les sexes tout au long de la vie.

## 1 Introduction

A life course perspective on sexual activity is gaining traction due to increased longevity and the recognized complex association between health and sexual activity. Informed by both biological and social factors, sexual aging has become a topic of interest and is even considered by some as a criterion for “successful” aging [1]. Embracing this perspective, Lindau and Gavrilova introduced the concept of sexually active life expectancy, highlighting the critical role of sexuality in overall health and well‐being [2]. This measure integrates sexual activity into traditional metrics of healthy life expectancy and related measures such as disability‐adjusted life years, which track progress in population health and assess health disparities among different groups. Using two national cohorts in the United States (US), Lindau and Gavrilova found that men generally had a longer sexually active life expectancy compared to women, largely due to a higher rate of partnership loss among women [2]. On the other hand, men experienced a greater reduction in years of sexual activity due to poor health compared to women [2].

The link between sexual activity and health in later life is well‐established, highlighting its importance for physical and emotional well‐being, leading some to consider sexual activity as a marker of healthy aging [3]. Although this perspective provides a more positive view of senior sexuality, it risks marginalizing older adults who do not fit the “sexy oldie” image [4] and may lead to an over‐medicalization of sexuality [3]. Moreover, focusing solely on partnered sexual activity fails to capture the changing nature of sexuality in later life, which adapts to physical limitations, living conditions, and societal norms [5, 6]. Fredriksen‐Goldsen advocates for a broader understanding of sexuality in later life by considering sexual quality of life, encompassing multiple dimensions and levels of satisfaction [5]. This broader approach aligns with the World Health Organization's definition of sexual health as “not merely the absence of disease, dysfunction or infirmity,” but as “a state of physical, emotional, mental, and social well‐being in relation to sexuality” [7, p. 5]. Supporting this broader concept, studies suggest that sexual satisfaction directly affects the quality of life in older adults, alongside the frequency of sexual activity and perceived importance of sexual activity in one's life [8, 9, 10]. This association is not only relevant to older adults but runs throughout the life course as shown in Moreau and colleagues' study of sexual lifestyles and health across the life course (this Special Issue) [11].

Recognizing sexual quality of life as a crucial aspect of sexuality, the sexual equity framework [5] fosters equitable experiences of sexual well‐being, agency, and satisfaction, while acknowledging the intersecting contextual, cultural, and individual influences—including factors such as age, gender, social class, race/ethnicity, and ability status—that shape sexual experiences. Gender differences are particularly notable; for instance, Lindau and Gavrilova show women aged 30 in the US can expect 4.7 fewer sexually active years than men, primarily due to a lack of partners [2], as women partner with older men who have a shorter life expectancy. Social norms historically and currently promote male sexuality, evident in the medicalization of male sexual dysfunction (e.g., Viagra), while limiting women's sexual expression, denying or neglecting their needs later in life [12]. These conditions influence sexual attitudes and behaviors throughout life [12] as well as individuals' lived experiences, with men frequently reporting higher levels of sexual well‐being than women [13].

These gender patterns have evolved and are context‐dependent, as suggested by Bajos and Bozon's work on sexuality in France, showing a smaller gender gap in sexual activity among individuals 50–69 years [13] than Lindau and Gavrilova in the United States [2]. In an analysis of 30‐year trends in France, published in this special issue, Bajos and colleagues also show that women of all ages consistently report greater sexual satisfaction than men in 2023 [14]. The study highlights a decreasing yet significant gender difference in sexual behaviors over time and generations, with men having a more diverse sexual repertoire, including more non‐partnered activities like masturbation, than women [14].

Building on Lindau and Gavrilova's concept of sexually active life expectancy, we aimed to explore how broadening this concept to include non‐partnered behaviors and subjective experiences of sexual life such as sexual satisfaction affects projections of sexual activity over the life course. Additionally, we examined the extent to which differences in partnership and health conditions explained gender differences in satisfying sexual activity in the last 12 months. We also conducted a stratified analysis of factors related to having a satisfying sexually active life over the past year among individuals aged 50 to 89, by sex.

## 2 Methods

### 2.1 Data Sources

This study draws on vital statistics and survey data to estimate sexually active life expectancy. Specifically, we used the French INSEE vital registration data to compute age group‐specific person‐years for the French population. We also used the CSF‐2023 (Contexte des sexualités en France 2023) national survey on sexuality in France to extract sexuality data [15]. The CSF‐2023 study includes a random sample of 21,259 participants aged 15–89 years who were selected using random digit dialing with a 34% response rate. Post‐stratification weights calibrated to 2023 census benchmarks (age, sex, region, education, household size) corrected for under sampling of adults aged 65–89 (only one in two were selected to reduce survey cost) and nonresponse bias, notably the underrepresentation of respondents without a high school diploma and under sampling of minors (full methodology described in Moreau et al. in this special issue) [15]. Weighting had only a modest effect on sexual activity measures, increasing by 1 percentage point reports of sexual debut before age 15 and decreasing by 1% reports of same‐sex and multiple partnerships. Participants responded to a 33‐min phone interview [15]. Adult respondents provided informed consent while minors provided parental consent and separately assented to participate. We restricted our analysis to men and women 20 to 89 years, who ever reported any sexual practice (*n* = 20,055) to estimate life tables of sexual activity over the life course. We further restricted to the 19,756 respondents with information on sexual satisfaction (511 missing, primarily due to survey dropout before reaching this final questionnaire item) when evaluating quality‐adjusted sexual life expectancy.

The questionnaire collected information on respondents' sociodemographic background, general health conditions, and various aspects of their sexual lives, including the number and sex of lifetime and 12‐month sexual partners, sexual practices in the last 12 months, sexual satisfaction at the time of the survey, and sexual dysfunction over the last 12 months. However, sexual dysfunction information was only collected from half of the sample who reported sexual activity in the last 12 months to reduce survey cost and therefore not considered in this analysis. The Committee of protection of people South East 1 (CPP Sud Est 1), the INSERM Ethics Evaluation Committee (CEEI‐IRB), and the Commission National Data Protection Authority (CNIL) approved this study.

### 2.2 Measures and Analysis

According to Lindau and Gavrilova, “sexually active life expectancy” refers to the “average number of remaining years an individual is expected to be sexually active” [2, p. 3]. This metric is calculated using Sullivan's method [16], which combines life table data based on census information with survey data on age‐specific prevalence of sexual activity. In our study, we utilized age‐ and sex‐specific population data and death counts from 2023 INSEE vital registration to compute age group‐specific person‐years by sex using life table techniques. We used CSF‐2023 data to assess the proportions of individuals involved in partnered and non‐partnered (masturbation) sexual activity in the past year, as well as the proportion satisfied with their current sexual lives. We assessed lifetime sexual activity by first asking respondents whether they had ever had sexual intercourse, and whether they had ever engaged in foreplay. Together, these items comprised our indicator of lifetime sexual practice. The term “sexual practice” was carried forward throughout the questionnaire to refer to both penetrative and non‐penetrative sexual activity. Sexual activity in the past 12 months was assessed by asking respondents “when they last had sexual practices with a partner”. This general measure was checked against practice‐specific questions on whether respondents had engaged in manual genital stimulation of a partner, oral sex, vaginal sex, or anal sex in the past 12 months. This cross‐check identified 164 cases where a practice‐specific question was reported but not captured by the general measure; in each case, the respondent's last partnered activity fell outside the 12‐month recall window (typically around 13 months), confirming their correct exclusion from the past‐12‐month indicator. Additionally, respondents were asked about masturbation in the last 12 months, to identify non‐partnered sexual practices in the last year. Sexual satisfaction, which we consider in this study as a quality metric of sexual life, was evaluated using a single question: “How satisfied are you with your current sexual life?”: with responses on a 4‐point Likert scale ranging from “Very satisfied” to “Not at all satisfied.” We dichotomized this information into two categories “satisfied” (combining very satisfied and satisfied) versus “dissatisfied” (combining dissatisfied and very dissatisfied). We calculated sexuality metrics according to five‐year age intervals, gender, partnership status, and health status. We determined partnership status by whether respondents had a cohabiting or non‐cohabiting partner at the time of the survey. We assessed health status through self‐reported measures used in the national health Barometer survey in France [17], which included functional disability and chronic illness. Participants indicated if they had any limitations in daily activities lasting at least 6 months due to a health problem and whether they had a chronic illness or health issue persisting for at least 6 months. We also asked if their current partner had any limitations in daily activities lasting at least 6 months.

In the second stage of the analysis, we conducted multivariate logistic regression models to examine whether partnership status at the time of the survey and health conditions explained differences in having a satisfying sexually active life in the last 12 months among individuals 15 to 89 years. Next, we conducted stratified analysis by sex to identify a broader set of social and health conditions related to having a satisfying sexually active life in the last 12 months by sex. We considered the following variables for this analysis; age group, sexual identity (heterosexual/non heterosexual including homosexual, bisexual, or other), highest level of education (< high school/high school graduation/bachelor/graduate education), perceived financial situation (stable/difficult or very difficult), migration status (migrant/non migrant), area of residence (large city/medium city/rural), and health/disability status. We conducted the same analysis among partnered individuals given the strong influence of partnership status on having a satisfying sexually active life. We conducted all analyses among the 19,756 respondents who reported any lifetime sexual activity and used weighted data to account for survey design and nonresponse.

## 3 Results

### 3.1 Characteristics of the Study Sample

The average age of respondents was 50.8 years, with 52% women and 48% men. More than three‐quarters of men (77%) had a partner at the time of the survey, compared to 74% of women. Men were more likely than women to report partnered sexual activity (82% vs. 72%) and non‐partnered sexual activity (64% vs. 43%). However, among sexually active respondents in the past year, women were more likely to report satisfaction with their sexual life (84% vs. 80%). Additionally, women reported higher rates of functional disability or chronic conditions than men (56% vs. 48%).

Patterns of partnerships and sexual experiences varied considerably over the life course (Table 1). The proportion of men with a current partner remained stable until the age of 75 while women's partnership status declined more rapidly after 50 years, resulting in a growing gender gap reaching 17 percentage points at 70–75 years and 44 points at 85–89 years. Gender differences in partnered sexual activity averaged 18 percentage points among respondents in their 60s, increasing to 29 percentage points in their 80s, while differences in non‐partnered activity fluctuated between 12 and 29 percentage points without a specific age trend. Among respondents who were sexually active in the past year, sexual satisfaction slightly decreased over time for both genders but dropped more sharply for men after 65 years (Table 1). Gender gaps in sexual satisfaction increased when respondents reached 70.

**TABLE 1:** Distribution of sexuality indicators and health according to sex and age among 20,055 men and women 20–89 years who ever reported any sexual activity in the CSF2023 survey.

	Partner		Unhealthy		Sexual activity last 12 months		Masturbation last 12 months		Sexual satisfaction^a^	
	Men	Women	Men	Women	Men	Women	Men	Women	Men	Women
Total	77.0	74.0	48.4	55.5	81.5	72.4	63.7	43.1	79.8	84.1
20–24	54.5	68.8	26.8	39.2	84.6	94.3	87.2	70.4	83.3	86.8
25–29	63.6	77.2	29.1	38.8	85.8	91.5	86.0	64.5	81.2	89.5
30–34	78.3	85.0	30.5	38.3	92.0	95.0	81.8	58.6	84.0	87.0
35–39	81.2	85.8	31.5	44.8	91.1	90.8	78.2	59.3	81.6	83.9
40–44	82.1	80.5	38.4	46.2	92.3	88.1	71.7	56.0	80.6	85.2
45–49	79.9	79.9	42.4	54.4	88.4	86.1	68.2	48.8	82.2	84.2
50–54	79.5	76.9	50.6	54.6	83.8	79.5	66.6	41.9	81.4	82.9
55–59	76.1	72.3	57.8	62.0	81.6	71.9	55.5	36.0	77.2	79.5
60–64	79.3	71.9	62.2	63.9	80.3	63.7	52.5	32.1	79.6	81.2
65–69	81.2	76.9	65.4	65.7	79.0	58.7	46.8	29.5	81.0	80.4
70–74	82.0	65.4	67.3	67.9	65.5	50.4	45.7	24.5	69.1	85.3
75–79	83.1	63.5	71.7	73.6	61.1	35.8	28.6	16.0	75.5	84.5
80–84	77.5	47.4	79.1	79.9	40.6	13.9	25.7	11.3	59.3	76.1
85–89	76.6	32.5	74.7	75.3	37.3	5.7	26.2	11.2	67.6	84.3

^a^ Estimated among respondents who reported any sexual activity in the last year.

In addition to sexual experiences, perceptions of health also varied over the life course with gradual increases in reports of functional disability or chronic illness by age in both sexes, although the gender gap favoring younger men disappeared when respondents reached their 60s (Table 1).

### 3.2 Sexually Active Life Expectancy

Patterns of sexual experiences, combined with gender mortality patterns, result in gendered patterns of sexually active life expectancies (Figure 1, Table 2). Under the conditions observed in 2023, our results indicate that a 20–24‐year‐old man has a sexually active life expectancy of 47.1 years, compared to 44.5 years for a 20–24‐year‐old woman, a gender gap of 2.6 years favoring men, despite a 4.6‐year difference in overall life expectancy favoring women (Figure 1, Table 2).

**FIGURE 1:**
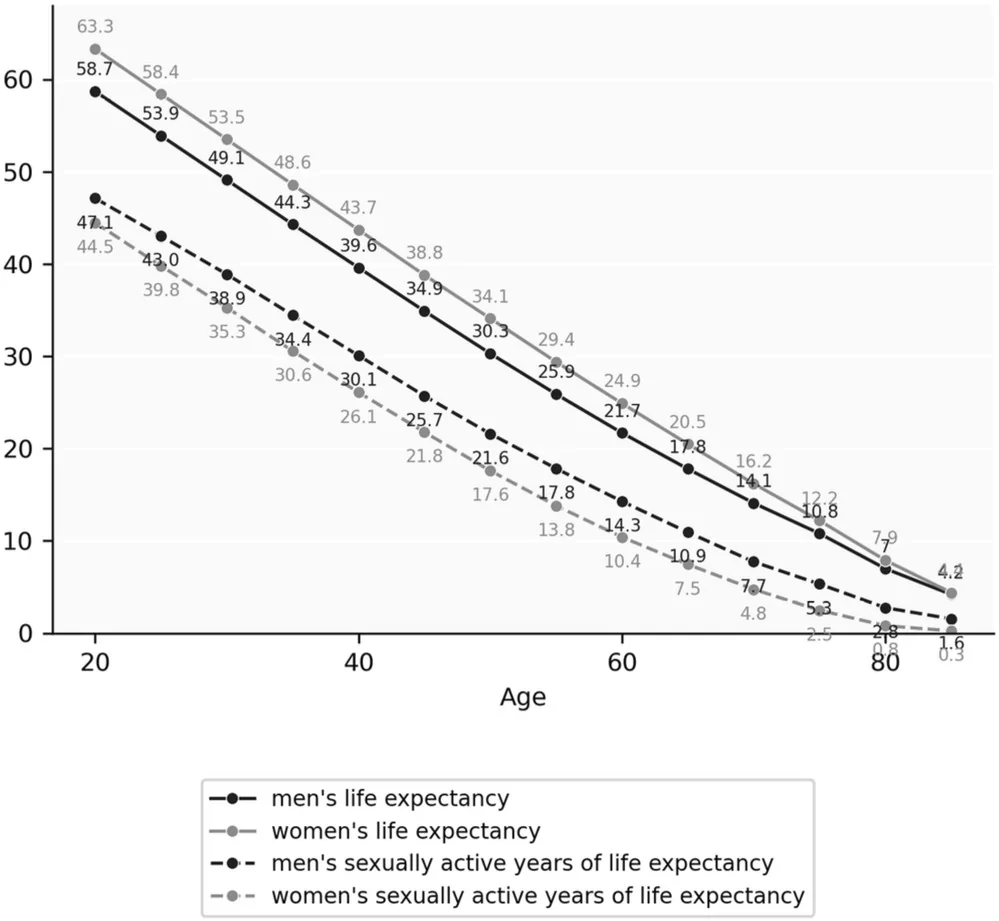
Life expectancy and sexually active life expectancy (partnered) by sex among participants of the CSF‐2023 study.

**TABLE 2:** Life expectancy and sexually active years of life expectancy, partnered only, including masturbation and adjusted for sexual satisfaction by sex among participants of the CSF‐2023 survey.

Age group	Men							Women						
	Life expectancy	Prevalence of sexual activity	Sexually active life expectancy	Prevalence of sexual activity + masturbation	Sexually active life expectancy including masturbation	Sexually active and satisfied	Quality adjusted sexually active life expectancy	Life expectancy	Prevalence of sexual activity	Sexually active life expectancy	Prevalence of sexual activity + masturbation	Sexually active life expectancy including masturbation	Sexually active and satisfied	Quality adjusted sexually active life expectancy
20–24	58.7	84.6%	47.1	98.2%	52.9	81.7%	41.9	63.3	94.5%	44.5	98.1%	48.9	85.1%	41.0
25–29	53.9	85.8%	43.0	98.0%	48.1	79.6%	38.0	58.4	91.5%	39.8	96.1%	44.1	85.9%	36.8
30–34	49.1	92.0%	38.9	98.1%	43.4	82.3%	34.1	53.5	95.0%	35.3	98.1%	39.3	85.3%	32.5
35–39	44.3	91.1%	34.4	98.4%	38.7	80.2%	30.1	48.6	91.0%	30.6	94.4%	34.5	79.1%	28.3
40–44	39.6	92.3%	30.1	98.5%	34.0	79.4%	26.3	43.7	88.1%	26.1	92.8%	29.8	78.9%	24.4
45–49	34.9	88.4%	25.7	95.9%	29.3	78.7%	22.5	38.8	86.1%	21.8	90.4%	25.3	76.0%	20.6
50–54	30.3	83.8%	21.6	93.6%	24.9	76.1%	18.9	34.1	79.5%	17.6	86.1%	20.9	71.2%	16.9
55–59	25.9	82.1%	17.8	91.2%	20.7	70.3%	15.4	29.4	71.9%	13.8	81.7%	16.9	64.6%	13.5
60–64	21.7	80.3%	14.3	91.0%	16.8	72.3%	12.4	24.9	63.7%	10.4	75.3%	13.0	60.8%	10.5
65–69	17.8	79.0%	10.9	87.6%	13.0	70.8%	9.4	20.5	58.7%	7.5	68.7%	9.5	54.8%	7.7
70–74	14.1	66.1%	7.7	80.4%	9.5	55.4%	6.5	16.2	50.4%	4.8	62.5%	6.4	52.4%	5.2
75–79	10.8	62.3%	5.3	70.2%	6.6	52.2%	4.4	12.2	35.8%	2.5	45.1%	3.6	37.1%	2.8
80–84	7.0	41.2%	2.8	58.6%	3.8	34.2%	2.3	7.9	13.9%	0.8	23.1%	1.6	16.5%	1.1
85–89	4.2	37.3%	1.6	48.9%	2.0	31.0%	1.3	4.4	5.7%	0.4	15.8%	0.7	11.6%	0.5

When we broaden the definition of sexual activity to include masturbation, the sexually active life expectancy at age 20–24 extends to 52.9 years for men and 48.9 years for women, increasing the gender gap to 4.0 years (Figure 2, Table 2). In absolute terms, the gender gap favoring men increases slightly until 35–39 years, and reduces thereafter, dropping to 3 years at 75–79 years. In relative terms, 20–24‐year‐old men can expect to spend 89% of their lives sexually active compared to 77% for women of the same age group (Figure 2, Table 2).

**FIGURE 2:**
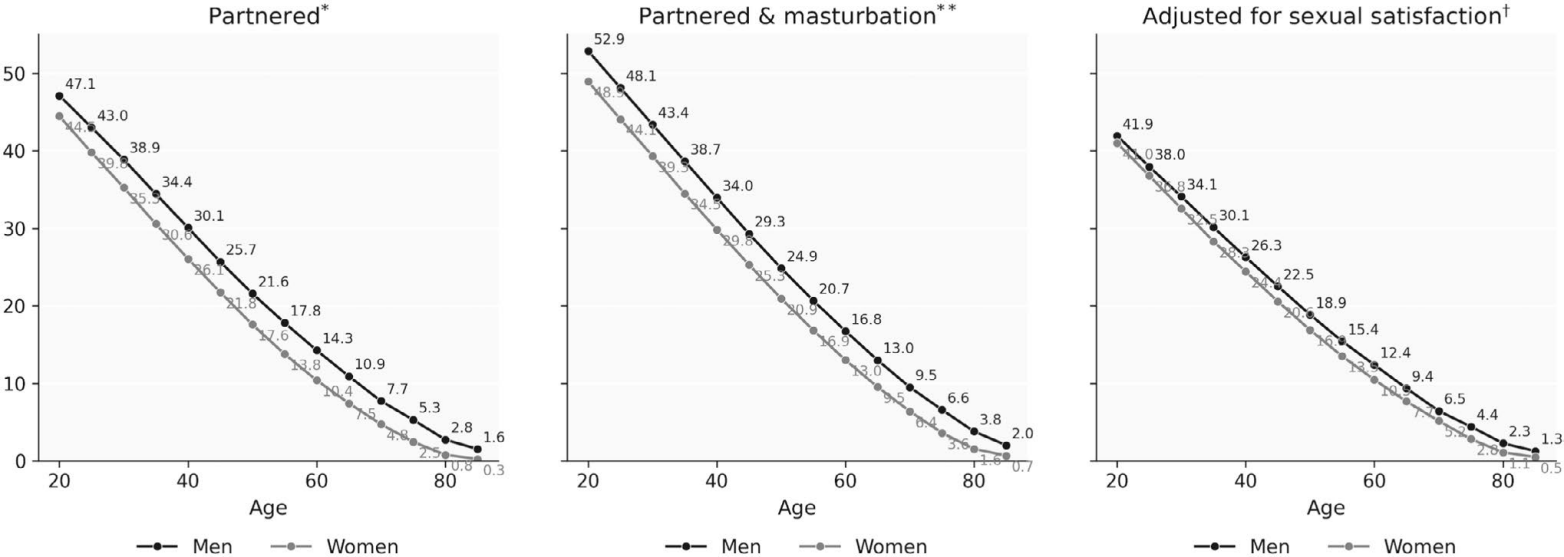
Sexually active life expectancy, partnered only, including masturbation and adjusted for sexual satisfaction by sex among participants of the CSF2023 survey. *Partnered: Sexually active years of life expectancy based on reporting sexual activity in the last year with a partner. **Partnered and masturbation: Sexually active years of life expectancy based on reporting sexual activity in the last year with a partner or masturbation in the last year. †Adjusted for sexual satisfaction: adjusted quality of sexually active years of life expectancy based on reporting sexual activity in the last year with a partner or masturbation in the last year and reporting a satisfying sexual life.

### 3.3 Quality‐Adjusted Sexually Active Life Expectancy

When factoring in sexual satisfaction as a criterion for the quality of sexual life, the quality‐adjusted sexually active life expectancy at age 20–24 decreases to 41.9 years for men and 41.0 years for women, as men aged 20–24 lose an average of 11 years of sexual life expectancy due to poor quality of their sexual experiences compared to 7.9 years for women (Figure 2, Table 2). The gender gap in quality‐adjusted sexually active years, which is 0.9 years for those aged 20–24, steadily increases, reaching 2.0 years for people 50–54 years, but declines thereafter (Figure 2, Table 2). In relative terms, a 20–24‐year‐old man can expect to spend 71% of his remaining years being sexually active and satisfied, compared to 65% for a 20–24‐year‐old woman.

Given sex differences in partnership and health status, especially after 50 years of age, we investigated the extent to which these conditions explain the gender gap in satisfying sexually active life among respondents 50–89 years. Results from multivariable regression models show a weakening of the gender gap in being sexually active and satisfied, narrowing from OR = 0.6 [0.6–0.7] after adjusting for age alone to OR = 0.8 [0.7–0.9] after adjusting for age, partnership status, sexual identity, disability, education, migration and health status. In stratified analysis by sex (Table 3), age, partnership status, disability, and health are the most significant correlates for both sexes. Other influential factors include education for women. These factors remain significant among partnered respondents.

**TABLE 3:** Sociodemographic and health correlates of reporting satisfying sexual activity in the last year among respondents 50–89 years by sex among participants of the CSF2023 survey.

		Men			Women		
		All	All	Partnered	All	All	Partnered
		% sexually active and satisfied	*n* = 4220	*n* = 3467	% sexually active and satisfied	*n* = 4667	*n* = 3273
Age (years)	50–54	76.1%			71.2%		
	55–59	70.3%	0.84 [0.65–1.09]	0.81 [0.58–1.11]	64.6%	0.79 [0.62–1.02]	**0.74 [0.54–1.01]**
	60–64	72.3%	**0.55 [0.41–0.74]**	**0.41 [0.29–0.58]**	60.8%	**0.55 [0.42–0.72]**	**0.48 [0.34–0.68]**
	65–69	70.8%			54.8%		
	70–74	55.4%	**0.25 [0.18–0.35]**	**0.20 [0.13–0.26]**	52.4%	**0.25 [0.18–0.35]**	**0.23 [0.15–0.33]**
	75–79	52.2%			37.1%		
	80–84	34.2%	**0.15 [0.08–0.29]**	**0.13 [0.06–0.34]**	16.5%	**0.12 [0.05–0.30]**	**0.03 [0.01–0.08]**
	85–89	31.0%			11.6%		
Sexual orientation	Heterosexual	66.9%			55.8%		
	Bisexual/homosexual or other	63.0%	1.35 [0.79–2.32]	0.60 [0.36–1.01]	46.0%	0.95 [0.57–1.60]	0.76 [0.35–1.66]
Partnership status	Partner with no functional disability	78.7%	**9.71 [7.5–12.56**]	**1.82 [1.43–2.32**]	76.8%	**11.43 [9.14–14.30**]	**2.01 [1.58–2.54**]
	Partner with functional disability	63.7%	**5.33 [3.99–7.13**]	Ref	56.3%	**5.52 [4.27–7.13**]	Ref
	No partner	29.6%	Ref		18.5%	Ref	
Education	> High school	63.2%	Ref	Ref	47.3%	Ref	Ref
	High school	66.0%	0.87 [0.66–1.14]	0.82 [0.60–1.11]	58.9%	1.18 [0.92–1.51]	1.05 [0.79–1.40]
	College education	71.1%	0.99 [0.78–1.25]	0.94 [0.72–1.22]	64.7%	**1.32 [1.07–1.63]**	1.25 [0.97–1.62]
	Graduate education	71.6%	0.98 [0.78–1.23]	0.96 [0.74–1.23]	70.2%	**1.53 [1.22–1.91]**	**1.57 [1.19–2.09]**
Migration status	Born French	65.4%	Ref	Ref	54.4%	Ref	Ref
	Migrant	68.8%	1.38 [0.85–2.25]	1.32 [0.76–2.28]	46.5%	0.75 [0.48–1.19]	0.96 [0.49–1.88]
Residence	Urban	59.9%	Ref	Ref	39.8%	Ref	Ref
	Rural	65.3%	1.18 [0.97–1.44]	1.19 [0.95–1.49]	55.1%	1.11 [0.92–1.34]	1.15 [0.92–1.45]
Health and disability status	Healthy	74.2%	Ref	Ref	65.0%	Ref	Ref
	Unhealthy	60.3%	**0.65 [0.52–0.80]**	**0.72 [0.57–0.92]**	48.3%	**0.64 [0.53–0.78]**	**0.55 [0.43–0.69]**

*Note:* Numbers in bold indicate statistical significance *p* < 0.05.

## 4 Discussion

Fifteen years after Lindau and Gavrilova first introduced the concept of sexually active life expectancy in the United States [2], our study builds upon this framework in a different cultural context—one characterized by more permissive sexual norms [18]. Our projections suggest that a 30‐year‐old resident in France experiences on average over 4 more years of sexually active life compared to a 30‐year‐old US resident, irrespective of sex (38.9 vs. 34.7 for men and 35.3 vs. 30.7 for women). The gap is not only explained by a longer overall life expectancy in France as compared to the United States, but also by a higher proportion of life spent being sexually active (79% vs. 72% for men 66% vs. 60% for women).

These estimates significantly underestimate the sexually active years of life by excluding masturbation, which increases sexually active years by 4.5 years for men and 4.1 years for women aged 30–34 in France. However, these figures likely still fall short of reality, due to the underreporting of masturbation among women due to social stigma [19, 20] and the growing importance of non‐genital forms of sexuality (touching, hugging, or kissing) in older age that are not all captured in our survey [6, 21]. Reports of masturbation have surged across generations, with more than a 30‐percentage point increase for women aged 50–69 between 1992 and 2023 and a 7–13 percentage point rise for men of the same age [14]. This trend suggests that non‐partnered sexual activity will increasingly influence estimates of sexually active life expectancy in the years to come.

Sexual practices alone do not capture the quality of sexual life, given rising sexual dysfunctions and reduced sexual satisfaction with aging [6, 22]. In our study however, sexual satisfaction only drops among sexually active men after 70 years of age. When factoring sexual satisfaction as a subjective positive dimension of sexuality, men in our study lose 11 years and women 8 years of quality‐adjusted sexually active life expectancy due to sexual dissatisfaction. This gap highlights the multiple facets of sexuality, which extend beyond mere behaviors to include elements of feelings and intimacy, which increasingly contribute to the quality of sexual life [5], including sexual satisfaction in later life. These features should be considered in future research to assess the quality of sexual life more comprehensively, along with other dimensions related to people's autonomy in engaging in sexual behaviors.

While these results provide valuable insights into the gendered dimension of sexual activity over the life‐course, several limitations warrant cautious interpretation. First, we recognize the constraints of the Sullivan method; cross‐sectional data do not capture changes in sexual activity patterns across an individual's lifetime. Our calculations to produce synthetic indicators do not account for generational shifts in sexual behavior, anticipated given the recent rise in non‐partnered sexual activity, especially among younger women, alongside a decrease in partnered activity [14]. Second, our estimates rely on self‐reported sexual activity, which can be perceived differently by men and women, leading to differential reporting [23]. Gendered perceptions of what constitutes a sexual partner contribute to the traditional gender gap in the reported number of lifetime sexual partners, where women typically count only those partners with whom they have engaged in sexual activity, while men often include all encounters [24]. The CSF‐2023 survey gathers information on sexual dysfunction only from half of the subsample of individuals who engaged in sexual activity in the past 12 months, limiting the scope for examining sexual dysfunction in this analysis. Finally, the survey excludes institutionalized individuals, representing 4% of individuals aged 60% and 10% after the age of 85 [25]. This exclusion leads to an overestimation of sexual activity among older adults.

Despite these limitations, this study enhances population‐level indicators of sexual health by adopting a life course approach and expanding the conceptual understanding of sexuality beyond partnered sexual behaviors. This indicator highlights notable gender differences in sexual experiences throughout the life course, which lessen when sexual quality is considered. These differences are expected to decrease further over time, due to recent changes in sexual behaviors among younger generations, particularly among women.

## Funding

This work was supported by French National Agency of Aids Research (ANRS), ECTZ117535, Santé publique France, French Ministry of Health, Institut national de la jeunesse et de l'éducation populaire, and Mission Interministérielle de Lutte Contre les Drogues et les Conduites Addictives.

## Data Availability

The data that support the findings of this study are available on request from the corresponding author. The data are not publicly available due to privacy or ethical restrictions.
